# Multiplexing in photonics as a resource for optical ternary content-addressable memory functionality

**DOI:** 10.1515/nanoph-2023-0406

**Published:** 2023-10-31

**Authors:** Yanir London, Thomas Van Vaerenbergh, Luca Ramini, Antoine Descos, Luca Buonanno, Jinsung Youn, Can Li, Catherine E. Graves, Marco Fiorentino, Raymond G. Beausoleil

**Affiliations:** Hewlett Packard Labs, 820 N McCarthy Blvd, Milpitas, CA 95035, USA; Hewlett Packard Labs, Hermeslaan 1A, 1831 Diegem, Belgium; Advanced Micro Devices Inc, Santa Clara, CA, USA; The University of Hong Kong, Hong Kong, Hong Kong

**Keywords:** energy efficiency, integrated photonics, silicon photonics, ternary content-addressable memory, time division multiplexing, wavelength division multiplexing

## Abstract

In this paper, we combine a Content-Addressable Memory (CAM) encoding scheme previously proposed for analog electronic CAMs (E-CAMs) with optical multiplexing techniques to create two new photonic CAM architectures—wavelength-division multiplexing (WDM) optical ternary CAM (O-TCAM) and time-division multiplexing (TDM) O-TCAM. As an example, we show how these two O-TCAM schemes can be implemented by performing minor modifications in microring-based silicon photonic (SiPh) circuits originally optimized for exascale interconnects. Here, our SiPh O-TCAM designs include not only the actual search engine, but also the transmitter circuits. For the first time, we experimentally demonstrate O-TCAM functionality in SiPh up to ∼4Gbps and we prove in simulation feasibility for speeds up to 10 Gbps, 10 times faster than typical E-TCAMs at the expense of higher energy consumption per symbol of our O-TCAM Search Engine circuits than the corresponding E-TCAMs. Finally, we identify which hardware and architecture modifications are required to improve the O-CAM’s energy efficiency towards the level of E-CAMs.

## 1 Introduction

The constant increase in demand for high-bandwidth low-latency applications has been driving the deployment of optical high-speed interconnects in modern cloud and high-performance computing (HPC) systems, due to the significant higher bandwidth and lower energy consumption compared to electrical links. Moreover, the continuous necessity to improve the energy consumption in other segments of datacenters and HPC systems have been pushing to allocate growing number of data processing tasks from CPU to memory module, to minimize latency and data movement [1, 2]. Content-addressable memories (CAMs) are a class of high-speed memory that search its entire content within a single cycle. This property is particularly handy for routers, switches, and SmartNICs in datacenters and HPCs, as it allows high-rate packet routing [3] and security analysis [4]. Recently, CAMs have been shown to be also useful in machine learning and deep learning applications [5–7]. Furthermore, numerous CAM designs have been developed, e.g., memristor-based [8], flash-based [9], and FeFET-based CAMs [10], that present continuous improvement in footprint and power consumption compared to previous generations. However, a content search in optical network applications requires optoelectronic conversion (OEC) as well as data-rate down-conversion because of the much lower speed of current electronic CAMs (E-CAMs), i.e., tens of gigabits in optical signal versus few of gigabits in search speed [11]. Even though these two routines are crucial for a content search, their energy consumption is not included in the energy metric of E-CAMs, thus creating the illusion of much lower energy performance than the true cost [12, 13]. To address this data rate discrepancy, an all-optical binary CAM (O-BCAM) cell, which stores data bit and compares it with a search bit using an optical flip flop and XOR gate, was proposed and experimentally demonstrated [14]. The design of this optical cell mimics the architecture of a typical E-CAM cell, but operates at speed of 10 Gbps, about 10 times faster than E-CAM cells. The functionality of this cell was extended to optical ternary CAM (O-TCAM) cell, which has a third search state of *don’t care*, in Ref. [15]. However, because these cells are based on InP coupled SOA-MZI switch, they have large footprint and energy consumption that limit their scalability. An alternative O-BCAM design, where its cells are based on microring modulators (MRMs), was proposed in Ref. [16]. Due to the small footprint and high wavelength selectivity of MRMs, this design is much more compact, energy-efficient, and scalable than the competing SOA-MZI based optical CAMs (O-CAMs). However, the cost of moving the data is not incorporated in this initial energy-efficiency analysis. Moreover, it is mentioned that a search state of *don’t care* can be added by eliminating the light at specific bit positions, but the additional hardware to execute this effectively is not discussed.

To overcome the data rate discrepancy between the optical signal and operating speed of E-CAMs as well as out of sight energy consumption issues, we developed two new O-TCAM architectures for Datacom. Specifically, our designs carries out the search word transmission and comparison to the stored words in the optical domain. Our proposed designs are a wavelength-division multiplexing (WDM) O-TCAM [17] and a time-division multiplexing (TDM) O-TCAM, where both utilize silicon photonics (SiPh) microrings for the core-operations of modulation and comparison. Multiplexing has a conspicuous benefit in photonics designs, such as SiPh microring-based circuits, compared to electronics due to the much higher channel count and aggregated data rate that result in extremely energy efficient devices [13]. In this work we study the role of the multiplexing on the energy consumption of the proposed O-TCAM designs. Importantly, while other circuit designs are possible, the proposed designs in the next two subsections have specifically been chosen with as they only have minimal changes compared to prior traditional HPC interconnect circuits [13], offering the potential for experimental validation (see Section 3). Unfortunately, as will be discussed in Section 5, this leads to some inefficiencies in energy consumption, but as will be shown with an example in Section 5.2, alternative choices can mitigate some of these issues in future work.

## 2 Proposed architectures

**Figure 1:**
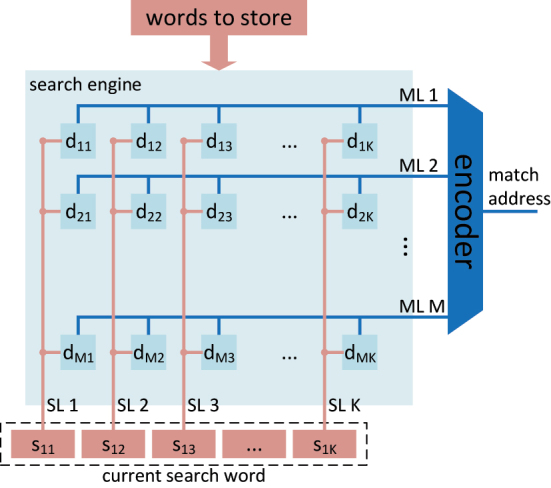
A conceptual diagram of a CAM with Search Engine size of *M* × *K*.

A conceptual diagram of a CAM is shown in Figure 1 [18]. The search word consists of *K* bits, where the *k*th bit is designated as *s*_*k*_. The search engine stores *M* words, where the *k*th bit of the *m*th stored word is designated as *d*_*mk*_. During a search routine, every one of the bits of the search word is distributed along its search line (SL), and compared with *M* bits, each belongs to a different stored word. The comparison results of the *m*th stored word are then combined at its match line (ML). In case that a ML identifies a match between the entire search word and its stored word, the encoder, in turn, specifies the match location.

We focus here on the subclass of ternary CAM (TCAM), where the bits can be *‘0’*, *‘1’*, or *X* (*don’t care*), thus allowing more search flexibility compared to binary CAMs (BCAMs), where only binary bits can be stored. The flexibility of searching for any stored value and comparing to any search value is typically implemented by merging two binary cells as in NOR-type CAM or adding a mask bit as in NAND-type CAM to form a single ternary cell [18]. In both design approaches, each of the stored symbols is encoded with two bits to represent one of the three states. Similar two-bit encoding schemes are used in memristive TCAMs, such as the encoding scheme presented in Ref. [7] that leverages the bitwise dot product operation to determine the matching state within symbol duration, i.e., **0** and **1** outcomes indicate a Match and a Mismatch, respectively. This encoding scheme is summarized in Table 1. This scheme suits well our SiPh platform, as will be presented in the following sections for both proposed O-TCAM architectures.

**Table 1:** Bitwise dot product based TCAM encoding scheme [7].

Search item		Stored item		Decision
Bits	Symbol	Bits	Symbol	
(*s*_1_, *s*_2_)	**s**	(*d*_1_, *d*_2_)	**d**	$\phi =\overset{2}{\underset{i=1}{\sum }}\;s_{i}\cdot d_{i}==0$
(*‘0’*, *‘1’*)	**0**	(*‘1’*, *‘0’*)	**0**	Match
(*‘0’*, *‘1’*)	**0**	(*‘0’*, *‘1’*)	**1**	Mismatch
(*‘1’*, *‘0’*)	**1**	(*‘1’*, *‘0’*)	**0**	Mismatch
(*‘1’*, *‘0’*)	**1**	(*‘0’*, *‘1’*)	**1**	Match
(*‘0’*, *‘0’*)	Search for **X**	(*X*, *X*)	**0** or **1**	Match
(*X*, *X*)	**0** or **1**	(*‘0’*, *‘0’*)	Compare to **X**	Match

The search (stored) symbol values are marked in bold to indicate that each of them consists of two search (stored) bits.

### 2.1 WDM O-TCAM

**Figure 2:**
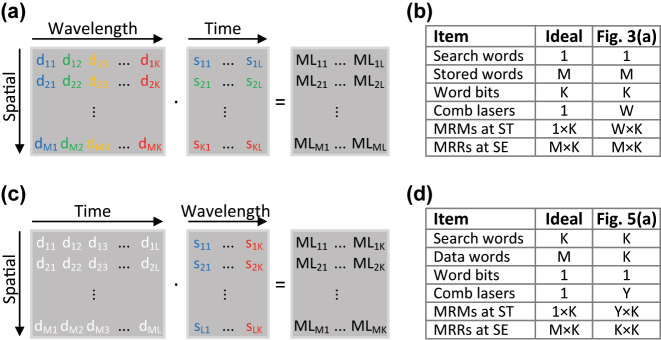
Conceptual diagrams of (a) WDM O-TCAM and (c) TDM O-TCAM architectures, which are based on matrix-matrix products. In the case of the TDM O-TCAM, the white color of the bits in the engine designated that the data words are encoded in multiple wavelengths, as shown in Figure 5(a). Scalability tables of (b) WDM O-TCAM and (d) TDM O-TCAM architectures. The total number of each of the items in the tables is determined at a single clock cycle.

**Figure 3:**
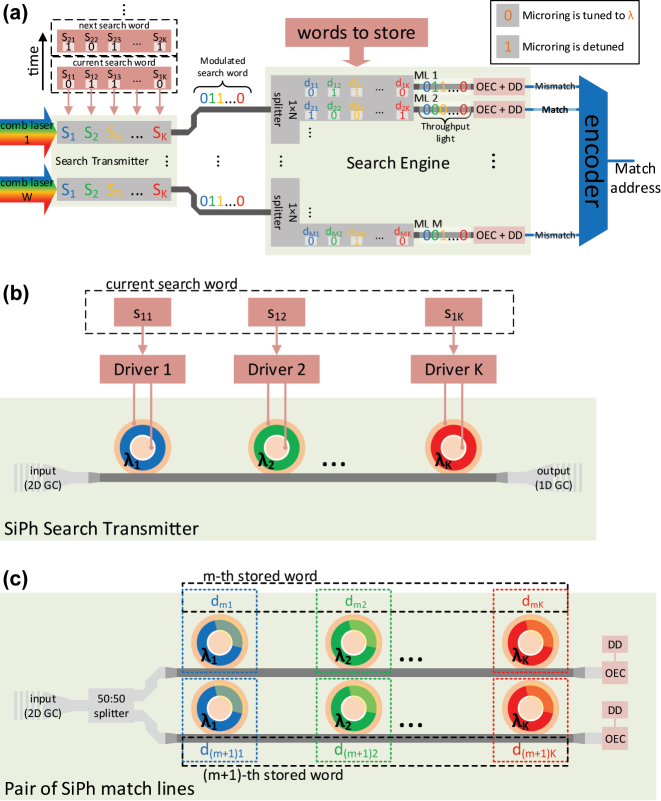
Microring-based WDM O-TCAM architecture. (a) A block diagram of the proposed architecture. (b) A schematic of one of the SiPh MRM-based transmitter circuits, located in the Search Transmitter. (c) A schematic of a MLs circuit with splitter size of 1 × 2, as an example, located in the Search Engine. *m* and (*m* + 1) are the indexes of the MLs, where *m* ∈ [1, *M* − 1]. GC, OEC, and DD are acronyms of grating coupler, optoelectronic conversion, and decision device.

Our WDM O-TCAM architecture generalizes the optical binary CAM (O-BCAM) in Ref. [16], following the ternary search states. Leveraging the bitwise dot product property of the encoding scheme in Table 1, we organized the proposed architecture in a matrix-matrix multiplication arrangement, as shown in Figure 2(a), where the bits of the search and stored words are wavelength-encoded. This configuration allows us to compare a search word vector of *K* bits with matrix of *M* stored words, each with *K* bits, as well as utilize SiPh microrings in designing the WDM O-TCAM. The block diagram of the proposed WDM O-TCAM architecture is shown in Figure 3(a), where the search dimensions and devices count are listed in the table shown in Figure 2(b). The Search Transmitter is responsible for the electro-optic conversion (EOC) and distribution of the search word of current matching quest round. It is worth to mention that the overhead of the EOC can be removed once the O-TCAM is fully embedded in the optical fabric. A comb laser is the light source because of its numerous evenly spaced lines as well as its small footprint, cost-effectiveness, and chip level integration [19, 20]. The Search Transmitter consists of $W=\frac{M}{N}$ SiPh WDM transmitters, each with *K* MRMs that are cascaded along the bus waveguide. This cascaded microring-based transmitter design is well established in Datacom and has been shown to be very effective in moving Terabit-class data with extremely low energy consumption performance [13, 21], important merits that are useful also for O-TCAMs. The maximum length of a search word is determined by the free-spectral ratio (FSR) of the MRMs and the channel spacing. A schematic of one of the SiPh WDM transmitter circuits is shown in Figure 3(b). Each of the MRMs is tuned to one of the error-free comb lines, which is being modulated by a bit of the search word, resulting in search channels (SCs), i.e., search lines in frequency domain. The wavelength-division multiplexed bits are distributed through a fiber array towards the SiPh Search Engine circuit, which has at its front-end a 1 × *N* splitter, where *N* is an even positive number. The splitter shares the search word with *N* MLs, each returns the Match state between the search word and the *m*th stored word. This sharing step is expected to reduce the total energy consumption due to the power allocation of the Search Transmitter devices between *N* stored words. However, the splitter size, *N*, is limited by the link budget performance as will be shown in Section 5. An example of a pair (*N* = 2) of MLs is shown in Figure 3(c). Each of the MLs consists of *K* cascaded microring resonators (MRRs), where their resonance is the wavelength of their counterpart at the Search Transmitter. In both the Search Transmitter and the Search Engine, the microrings are organized in pairs to establish a symbol as defined in Table 1. At the Search Engine, the multiplexed bits are being “re-modulated”, here passively filtered, by their counterpart *K* bits of the *M* stored words. This re-modulation step is equivalent to a dot product between the search and stored signals. At the last stage, the re-multiplexed signal of a specific ML is converted to an electrical signal through photodetector (PD), the matching state is determined by a decision device (DD), and then the match address is presented at the output of the encoder. In this paper we use SiGe avalanche photodiodes (APDs) for the OEC step due to its high sensitivity and speed as well as low bias compatibility and CMOS compatibility, qualities that were found to be beneficial for performance and integration density in SiPh interconnect systems [22, 23] and are also desirable for O-CAMs. The OEC and DD are placed at the through port of the last MRR because of the much lower losses compared to the drop port. To make a decision whether the search and stored words are matched or not, a transimpedance amplifier (TIA) that converts from PD output current to voltage signal and a low-power comparator, which detects Match/Mismatch, are used after the PD. To minimize the power consumption, an inverter-based TIA configuration [24] is widely used. Moreover, a strong-arm latch comparator is popular in energy-efficient designs due to its zero static power consumption [25].

**Figure 4:**
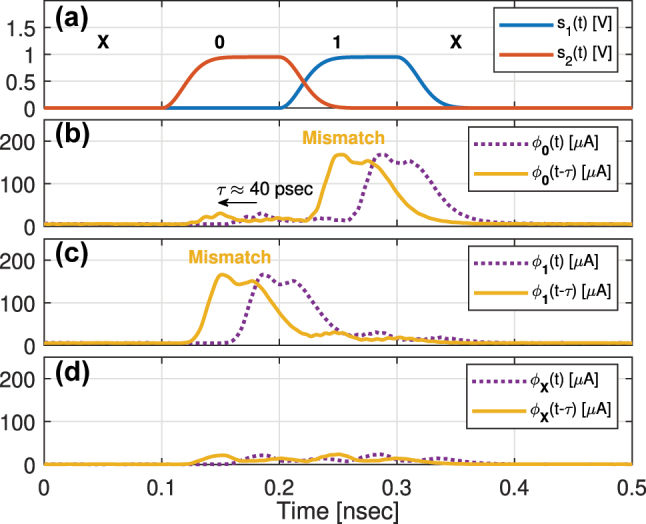
The simulation results of a two-bit WDM O-TCAM circuit. (a) The first and second search bit signals, *s*_1_(*t*) and *s*_2_(*t*). The search sequence is indicated above the search signals. (b)–(d) The shifted match signal (continuous) at the APD output, when compared to a stored symbol of **0** (subplot b), **1** (subplot c), or **X** (subplot d). The Mismatch states are indicated above the shifted match signals.

We simulated in Lumerical INTERCONNECT a 2-bit WDM O-TCAM, which allows us to execute a single symbol content search at each clock cycle, to demonstrate the operation of the proposed architecture in the time-domain. We modelled a Search Transmitter with two cascaded PIN MRMs and a Search Engine with two cascaded microring filters, where an APD is placed at the through port of the second ring. This allows us to map all the combinations listed in Table 1 through three experiments, where the search sequence is **X01XX**, a six words series, and the stored word is **0**, **1**, or **X**. The most- and least-significant bit sequences, *s*_1_(*t*) and *s*_2_(*t*), both construct the search words series, have a pulse shape of non-return-to-zero (NRZ) with rise and fall periods of 30 % of the bit duration and data rate of 10 Gbps. The first and second MRMs are driven by *s*_1_(*t*) and *s*_2_(*t*), respectively. The model of the MRMs is taken from the process design kit (PDK) we developed, as part of the Exascale Computing Project, for our research on exascale computing interconnects [13, 26, 27]. The driving signals, *s*_1_(*t*) and *s*_2_(*t*), are shown in Figure 4(a). The power of each of the laser lines is set to −6 dBm and the resonances of the first and second MRMs are 1309.2 and 1309.8 nm, respectively, and their Q and extinction ratio (ER) are 15 K and 13 dB. The Q and ER of the Search Engine rings, which operate as notch filters, were selected to be 6 K and 15 dB to ensure high attenuation of the transmitted modulated light at a Match state, which leads to high-contrast between Match and Mismatch states. In both the transmitter and engine, the amplitude of ‘1’ is 0.95 V, which results in resonance shift of 0.3 nm. The match signal, *ϕ*_**d**_(*t*), where the subscript **d** is the value of the stored word, is measured at the output of the APD [22] from our exascale PDK. The comparison results between the search sequence and the stored word are shown in Figure 4(b) (**d** = **0**), 4(c) (**d** = **1**), and 4(d) (**d** = **X**). The match signals (continuous) are shifted by *τ* to compensate for the delay resulting from the APD. The results show that in the three cases the match signal follows the decision scheme in Table 1 as expected. Moreover, the contrast between Match and Mismatch states depends on the ER of the ring filters, specifically the small APD current at Match state is minimized when all the rings block the light of all search bits as shown for **d** = **X**.

### 2.2 TDM O-TCAM

**Figure 5:**
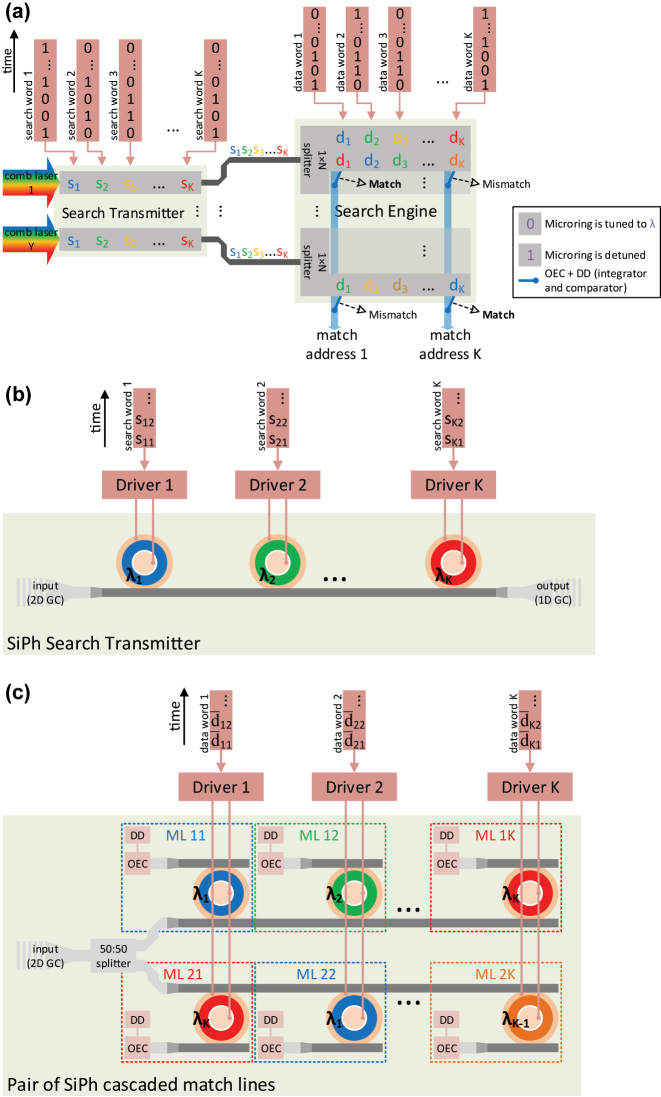
MRM-based TDM O-TCAM architecture. (a) A block diagram of the proposed architecture. (b) A schematic of one of the SiPh MRM-based transmitters, located in the TDM Search Transmitter. (c) A schematic of the 1-st and 2-nd cascaded MLs circuit, located in the TDM Search Engine. The bar above *d*_*i*,*j*_ indicates inverted bit value, required to accommodate the enecoding scheme when the match signal is measured at the microring drop port. GC, OEC, and DD are acronyms of grating coupler, optoelectronic conversion, and decision device.

The TDM O-TCAM architecture utilizes as well a cascaded SiPh microrings structure to execute a matrix-matrix multiplication, where the bits of the search and data words are time-encoded, as shown in Figure 2(c). The white color of bits indicates that in the proposed design each of the search words is compared with all the data words at the engine. A block diagram of the proposed TDM O-TCAM is shown in Figure 5(a), where the search dimensions and devices count are listed in the table shown in Figure 2(d). The number of channels, which determined by the FSR and channel spacing, sets the word count. Therefore, the Search Transmitter distributes simultaneously *K* search words towards the Search Engine, which in turn compares these words with *K* data words. The number of data words can be increased beyond *K* by attaching additional Search Engine units. Each pair of bits in the search and data words represent a symbol as defined in Table 1. Similarly to the WDM O-TCAM architecture, the Search Transmitter and Search Engine consist of $Y=\frac{K}{N}$ and *K* circuits of *K* cascaded SiPh MRMs along a bus waveguide, respectively. A schematic of one of the cascaded MRM-based transmitter circuit is shown in Figure 5(b). Every one of the MRMs in a transmitter circuit is tuned to one of the error-free comb lines, which is being modulated sequentially by the bits of a search word, i.e., at the *i*th unit interval, the bits located at the *i*th position of the search words are being transmitted through a fiber array towards the Search Engine device. The MRMs in the Search Engine are organized in a *K* × *K* array. Figure 5(c) shows a schematic of the first two rows of *K* cascaded MRMs. The resonance order of the MRMs in one row is shifted by one seat in the following row as demonstrated in Figure 5(a). With this resonance arrangement each of the search words is compared with all the stored words. To determine a matching state, an APD followed by a DD is located at the output of each MRM in the Search Engine. A match occurs when the integral over the word duration of the re-modulated signal is **0**, therefore each MRM in the Search Engine functions as an ML. The DD circuit can be realized by incorporating a TIA, an integrator, and a comparator. Once APD output current signal is converted to voltage signal by TIA, the voltage signal can be integrated over the word duration. Then, the integrated signal can be compared with a specific threshold level by the comparator.

**Figure 6:**
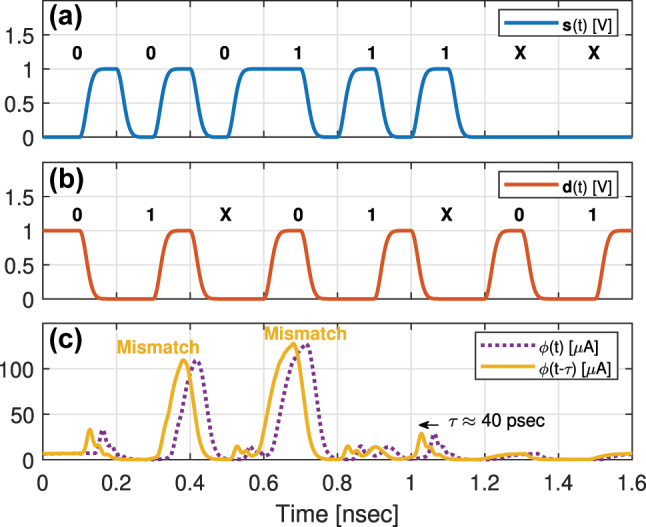
The simulation results of a TDM O-TCAM circuit of a single search word and a single data word. (a) The search signal, **s**(*t*). The search sequence is indicated above of the signal. (b) The data signal, **d**(*t*). The data sequence is indicated above of the signal. (c) The shifted match signal (continuous) at the APD output. The Mismatch states are indicated above of the shifted match signal.

To demonstrate the time-domain operation, we simulated in Lumerical INTERCONNECT a TDM O-TCAM circuit, where its Search Transmitter and Search Engine have a single PIN MRM, taken from our exascale PDK. The resonance, Q, and ER of these MRMs are 1309.5 nm, 15 K, and 13 dB. The power of the laser, which is connected to the input of the MRM of the Search Transmitter, is −6 dBm. The search and data sequences are **000111XX** and **01X01X01**, respectively, which allows us to map all the combinations listed in Table 1. The search signal, **s**(*t*), and data signal, **d**(*t*), have data rate of 10 Gbps and pulse shape of NRZ with amplitude of 1 V and rise and fall periods of 30 % of the bit duration. The search and data signals are shown in Figure 6(a) and (b), respectively. The match signal, *ϕ*(*t*), is measured at the output of our APD [22] and connected to the drop port of the MRM located at the Search Engine. The match signal is shown in Figure 6(c). We shifted *ϕ*(*t*) by *τ* to compensate for the delay resulting from the APD. The match signal in this experiment follows the decision scheme in Table 1 as expected. The small spikes that can be observed at the Match state are due to the dynamic of light within the microring cavity.

## 3 Experimental demonstration

**Figure 7:**
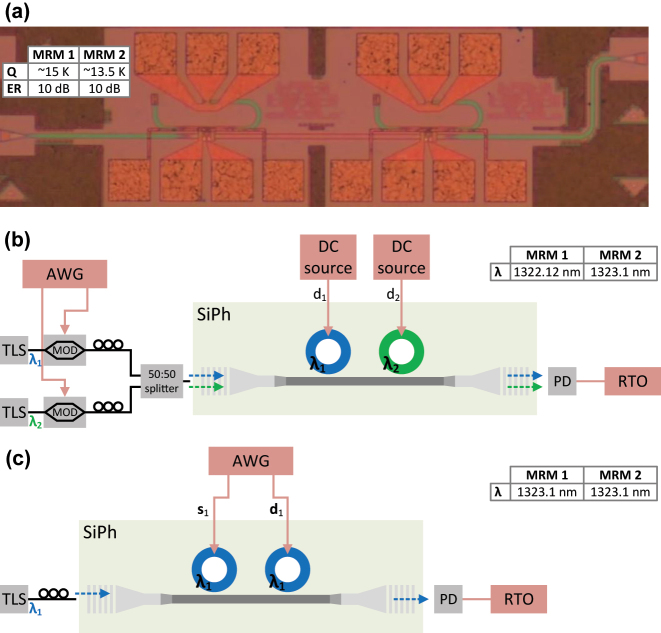
Proof of concept of the proposed O-TCAM architectures. (a) The two cascaded MRMs device, which was manufactured in CEA-LETI. (b) The experimental setup of the 2-bits WDM O-TCAM architecture. (c) The experimental setup of the 1-word TDM O-TCAM architecture.

**Figure 8:**
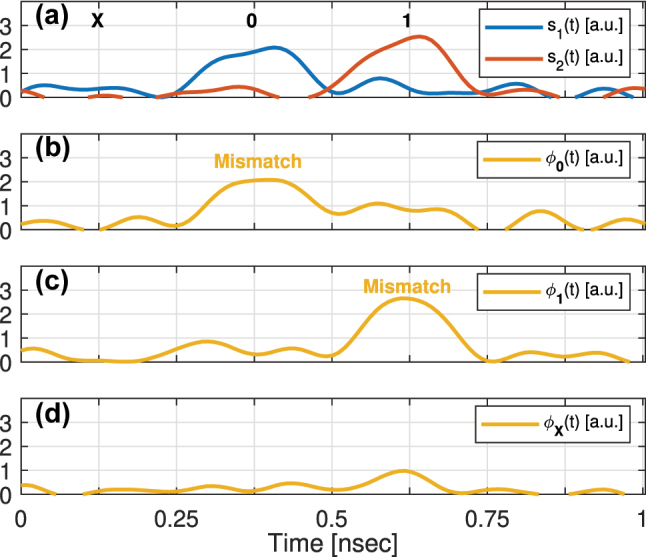
The experimental results of a 2-bits WDM O-TCAM architecture. (a) The first and second search bit signals, *s*_1_(*t*) and *s*_2_(*t*), which are measured by the RTO, and above them the search sequence is indicated. (b)–(d) The match signal, which is measured by the RTO, when compared to a stored symbol of **0** (subplot b), **1** (subplot c), or **X** (subplot d). The Mismatch events (post analyzed, threshold ∼1.5a.u. ) are indicated above the corresponding events in the match signal time traces.

We validated the operation of the proposed WDM and TDM O-TCAM architectures through proof-of-concept experiments. In these experiments, we used a test structure of two cascaded MRMs located on a SiPh wafer that was manufactured in CEA-LETI [28]. A photo of this device is shown in Figure 7(a), where the MRMs parameters are specified in the table. The setup for the analysis of the WDM O-TCAM design is shown in Figure 7(b). In this experiment, the cascaded microrings circuit operates as a 2-bit Search Engine, where the voltages applied by the DC sources set the data to store, i.e., **0**, **1**, or **X**. The lines at 1322.12 and 1323.1 nm, which are generated by the tunable laser sources (TLSs), each with power of 6 and 8 dBm, respectively (due of the unbalanced system losses), are being externally modulated by a preset sequence of 2-bit search words using 40 GHz Intensity Modulator1IXBlue MX1300-LN-40. (IM). The use in external modulators is a compromise aimed to demonstrate the proposed microring-based WDM O-TCAM architecture, which requires four microrings for 2-bit design, with the available test structure of two cascaded microrings. The 3.984 Gbps search sequence of **X01XX** is being generated by an 65 GSa/s arbitrary waveform generator2Keysight M8195A 65 GSa/s AWG. (AWG). Each of the search signals, *s*_1_(*t*) and *s*_2_(*t*), is measured at the output of an external PD3Thorlabs DX50AF Single Mode Ultrafast Detector Module. connected to the exit port of the SiPh circuit by a real-time oscilloscope4Tektronik MSO64B (8 GHz bandwidth) Mixed Signal Oscilloscope. (RTO) when the microrings are off-tuned. These measured signals are shown in Figure 8(a). The match signals, *ϕ*_**d**_(*t*), for **d** = **0**, **d** = **1**, and **d** = **X** are shown in Figure 8(b)–(d), respectively. In all three cases, the match signal follows the decision scheme presented in Table 1.

**Figure 9:**
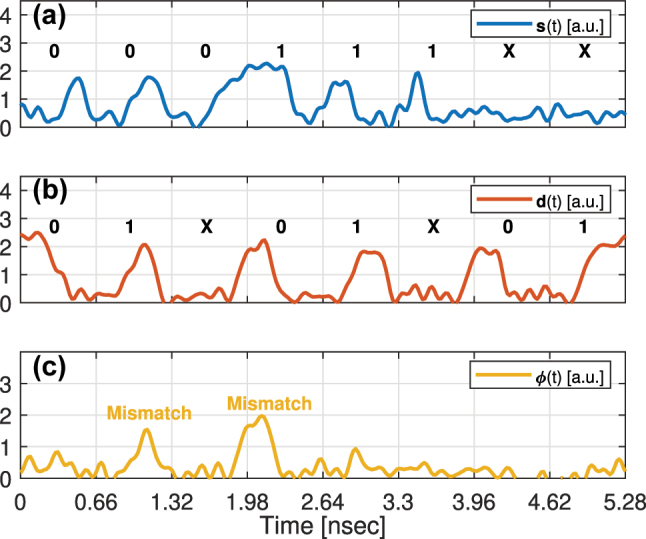
The experimental results of a 1-word TDM O-TCAM architecture. (a) The search signal, **s**(*t*), measured at the RTO, and above it the search sequence. (b) The data signal, **d**(*t*), measured at the RTO, and above it the data sequence. (c) The match signal, *ϕ*(*t*), measured at the RTO. The events corresponding to a Mismatch state (post analyzed, threshold ∼1.5a.u. ) are indicated in the *ϕ*(*t*) traces.

In the experiment of the TDM O-TCAM design, the first MRM operates as a 1-word Search Transmitter and the second MRM operates as a 1-word Search Engine. The setup is shown in Figure 7(c), where the MRMs are tuned to 1323.1 nm and the same AWG, PD, and RTO are used. The search sequence of **000111XX** and data sequence of **01X01X01**, respectively, are generated by the AWG and beat at 3.03 Gbps. The measured search and data signals, which are measured at the output of the PD by a RTO when the microrings are off-tuned, are shown in Figure 9(a) and (b). Afterwards, we compared the search and data signals using the 1-word TDM O-TCAM setup. The match signal, *ϕ*(*t*), is shown in Figure 9(c), and it can be observed that the received signal follows the decision scheme in Table 1.

## 4 Noise analysis

The size of the proposed O-TCAM architectures is limited by the FSR and the noise. While the FSR determines the maximum number of wavelength channels, *K*, the noise, on the other hand, dictates both the number of channels and the splitter degree, *N*, which allow us in the proposed architectures to share the energy cost of the drivers and heaters of the Search Transmitter. Specifically, the noise determines the required number of photons at the PD to achieve a specific signal to noise ratio (SNR) that guarantees the match decision quality, similar to how SNR determines the bit error rate (BER) in Datacom interconnects. The photon count, after taking into account the link losses, determines the maximum values of *N* and *K*, i.e., splitter size and microring count, at given comb line power. We evaluate here the scaling limitation of the proposed O-TCAMs due to noise, using similar methodology as in [29], which was developed for matrix-vector multiplication. At the full-scale of the WDM O-TCAM we have a matrix of *W* × *K* of stored words multiplied with a search word matrix of *K* × *L*, whereas in the TDM O-TCAM we have a multiplication between data word matrix of *K* × *L* and search word matrix of *L* × *K*. As our interest here is in a case study of matrix-vector product, we degenerated the WDM O-TCAM to a single clock cycle, i.e., a search word matrix of *K* × 1, and set the stored word matrix to *N* × *K*, which focuses the scaling evaluation on a single engine circuit of *N* stored words, enabled by the 1 × *N* splitter at its input. A similar simplification is done for the TDM O-TCAM—the Search Transmitter transmits a data word vector of *L* × 1 and the data matrix at the engine has a size of *N* × *L*. This is carried out by modulating *N* data words over the same resonance wavelength as the search word, which is allowed because of the spatial multiplexing enabled by the 1 × *N* splitter. To simplify the notation in the following model, we designate *D* as the number of symbols within a word, i.e., for WDM O-TCAM $D=\frac{K}{2}$ and for TDM O-TCAM $D=\frac{L}{2}$ .

Given the photon count in a unit interval of a search bit is ${\hat{s}}_{j}$ , where *j* is the bit index, then the total photon count constructing the match signal for the *i*th word is:

(1) $$\Phi_{i}=\underset{j}{\sum }{\hat{d}}_{i,j}{\hat{s}}_{j},$$

where the matrix $\hat{d}$ encodes the data words and correspondingly converts the photon count in $\hat{s}$ to the match vector Φ_*i*_. In this work, a SiGe APD, taken from our exascale PDK, is responsible for the optoelectronic conversion in the proposed O-TCAMs due to the SNR improvement when the APD operates at the avalanche regime [22]. In Refs. [22, 30], it is shown that the APD noise, comprising shot noise and dark current, dictates the APD sensitivity floor at avalanche operation mode. As a result, the total photon count is modified to [31]:

(2) $$\Theta_{i}=\Phi_{i}+\eta_{s,i},$$

where $\eta_{s,i}∼N\left(0,\sqrt{\Phi_{i}}\right)$ is a Gaussian random variable with zero-mean and variance of $\sqrt{\Phi_{i}}$ , where it is assumed that the contribution of the dark current to the total noise is significantly lower compared to the shot noise [32]. The total number of photons within the match signal depends on the mismatch gain, *H*_M_, which is the Hamming distance between Match and *h*th Mismatch states, where *h* ∈ *H*_M_, i.e., it equals to the number of mismatch events, therefore we can write that:

(3) $$\Phi_{i}=n_{\text{MAC},1}H_{\text{M},i},$$

where *n*_MAC,1_ is the number of photons for a single multiply-accumulate (MAC) operation, when a single mismatch event occurred and after considering the link losses. Because the electrical SNR scales with the square of the photocurrent, the SNR can be written as:

(4) $$SNR={\left(\frac{\Phi_{i}}{\sqrt{\Phi_{i}}}\right)}^{2}=n_{\text{MAC},1}H_{\text{M},i}.$$

This result shows that the electrical SNR scales with *n*_MAC,1_*H*_M,*i*_, which means that the SNR is effected not only by the number of photons through the link budget performance, but also by the Hamming distance. Moreover, we designate the number of photons for no mismatch events and one mismatch event within the match signal as Φ_*i*,0_ and Φ_*i*,1_, which is equal to *n*_MAC,1_. Because max *H*_M,*i*_ = *D*, then max Φ_*i*_ = Φ_*i*,1_*D*, however, a single mismatch event is already sufficient to lead to a Mismatch state for the full word, therefore the decision threshold is placed between Φ_*i*,0_ and Φ_*i*,1_.

This analysis describes the impact on the SNR performance due to shot noise, however, in case that non-avalanche mode of operation is considered for the APDs used in our exascale SiPh PDK, other noise mechanisms, such as TIA thermal noise, should be added in order to evaluate the link budget and specifically the required comb line optical power [29, 33]. Nonetheless, the conclusion of this paragraph that the decision threshold for a Match/Mismatch of a word lies between Φ_*i*,0_ and Φ_*i*,1_ still holds, and we can use the sensitivity of the APDs as a baseline to which the desired signal strength needs to be compared to obtain a certain BER, which will in turn determine how many photons are required at the laser. This is the procedure we will apply in the energy consumption discussion in the next section.

## 5 Energy analysis

The power consumption in the proposed designs happens in the comb source, drivers, microring heaters, OECs, and DDs. In the case of the TDM O-TCAM architecture, an additional local memory is required to store the data words. This can be done with DRAM, but due to its high access energy consumption of tens of pJ/bit [34], an energy efficient alternative would be SRAM, which consumes only a few fJ/bit. A 1 fJ/bit SRAM was demonstrated [35] at 10 MHz. An SRAM with much faster access speed of 935 MHz was shown in Ref. [36], but has energy consumption of 32.5 fJ/bit. Assuming that *l* × *K* bits of the *K* data words, which are loaded into the drivers of the search engine, are retrieved in parallel from the SRAM memory, the ∼1GHz operation of the SRAM can support the 10 Gbps data rate if *l* > 10. A *l* × *K* memory buffer, located close to the drivers, is needed in order to relay the *K* data words, retrieved from the SRAM to the engine. A flip flop (FF) in 28 nm, a consolidated technological CMOS node which is widely used for prototyping emerging accelerators chips [37, 38], can operate with a 1 ns clock period and consume 10 fJ/cycle. Therefore, both the SRAM and the flip-flop memory add 85 fJ/sym (42.5 fJ/sym) for the energy consumption of TDM O-TCAM (O-BCAM) at each comparison clock cycle. To compare fairly between the energy consumption of the two proposed architectures, we considered the energy consumption of comparing a single search word with *N* stored (data) words, which is using our proposed encoding equivalent to a matrix-vector product [7]. For the WDM O-TCAM design, the energy consumption during a single clock cycle of a Search Transmitter with a *K*-bits search word that is compared to *N* stored words in the Search Engine, normalized over the number of bits, number of words, and the Match/Mismatch decision speed, *R*_*b*_, is:

(5) $$\begin{array}{ll} E_{\text{ST}}^{\left(WDM\right)} & =\frac{\rho NKP_{\text{cl}}+K\left(P_{\text{dr}}+P_{\text{ht}}\right)}{NKR_{b}}\\ & =\rho E_{\text{cl}}+\frac{E_{\text{dr}}+E_{\text{ht}}}{N}, \end{array}$$

where *P*_cl_ is the minimal optical power of a comb line that is required to detect a Match/Mismatch at the PD (see SNR discussion in Section 4) and depends on the losses in the optical link between the laser and PD, e.g., excess losses of encountered splitters and insertion loss of microrings. $\rho =\frac{1+EP}{WPE}$ is the effective wall-plug efficiency (EWPE) that links between the optical power of the useful lines to the total comb power [23, 39]. The WPE stands for the wall-plug efficiency of the laser and EP stands for the rate of the excess power of lines within the usable comb bandwidth, *BW*_comb_, above *P*_cl_ as well as comb power outside *BW*_comb_. *P*_dr_ is the power of the modulator driver, which operates at a data rate of *R*_*b*_, and *P*_ht_ is the power of a microring heater, which is required to compensate for fabrication deviations and tune the microring to its target resonance wavelength. *E*_cl_, *E*_dr_, and *E*_ht_ are the energy consumption of a single comb laser wavelength line, driver, and microring heater in the Search Transmitter during a single clock cycle, i.e., per compared bit. The maximum energy consumption of a Search Engine with *N*-stored words, normalized over the number of bits, number of words, and decision speed, is:

(6) $$\begin{array}{ll} E_{\text{SE,max}}^{\left(WDM\right)} & =N\frac{K\left(P_{\text{ht}}^{d,\max}+P_{\text{ht}}\right)+P_{\text{dd}}^{\left(WDM\right)}}{NKR_{b}}\\ & =E_{\text{ht}}^{d,\max}+E_{\text{ht}}+\frac{E_{\text{dd}}^{\left(WDM\right)}}{K}, \end{array}$$

where $P_{\text{ht}}^{d,\max}$ is the maximum power of the heater to store the data **d**, i.e., all the bits of the stored word in the engine-side are *‘1’*, $P_{\text{dd}}^{\left(WDM\right)}$ is the power of the TIA and comparator needed to detect a Match/Mismatch as explained in Section 2.1. $E_{\text{ht}}^{d,\max}$ , *E*_ht_, and $E_{\text{dd}}^{\left(WDM\right)}$ are the correspond energy consumption of these power terms per clock cycle.

In the case of TDM O-TCAM, we perform similar simplification as in Section 4, where the Search Transmitter transmits an *L*-bit data word, which is being compared with *N* data words of a length of *L* bits, where *L* is an index of a clock cycle. Then the energy consumption of the TDM Search Engine, after normalizing over the number of bits, number of words, and decision speed, is:

(7) $$\begin{array}{ll} E_{\text{ST}}^{\left(TDM\right)} & =\frac{\rho NP_{\text{cl}}+P_{\text{dr}}+P_{\text{ht}}}{N\cdot \frac{R_{b}}{L}\cdot L}\\ & =\rho E_{\text{cl}}+\frac{E_{\text{dr}}+E_{\text{ht}}}{N}. \end{array}$$

As we have here a single search word transmission then only one comb line is required. Moreover, the Match/Mismatch decision for TDM O-TCAM can be done in principle at the end of the comparison sequence, i.e., at speed of $\frac{R_{b}}{L}$ . The energy consumption of a Search Engine with *N* data words, each with *L* bits, is:

(8) $$\begin{array}{ll} E_{\mathrm{SE}}^{\left(TDM\right)} & =N\frac{P_{\text{mr}}+P_{\text{dr}}+P_{\text{ht}}+P_{\text{dd}}^{\left(TDM\right)}}{N\cdot \frac{R_{b}}{L}\cdot L}\\ & =E_{\text{mr}}+E_{\text{dr}}+E_{\text{ht}}+E_{\text{dd}}^{\left(TDM\right)}, \end{array}$$

where *P*_mr_ includes the average power required to read from the SRAM and update a FF memory unit with a new bit of a data word. $P_{\text{dd}}^{\left(TDM\right)}$ includes the power of the TIA, comparator, and integrator needed to detect a Match/Mismatch as described in Section 2.2. *E*_mr_ and $E_{\text{dd}}^{\left(TDM\right)}$ are the corresponding energy consumption of these power terms per clock cycle. When comparing both energy consumption models for the circuit designs proposed in Section 2, it can be observed that while the energy consumption of the Search Transmitter is the same for both architectures, the Search Engine energy consumption of the TDM O-TCAM circuit is expected to be higher compared to the WDM O-TCAM circuit. We identify three reasons for this difference: (1) a difference in the microring’s static power consumption in the Search Engine, (2) a difference in the power consumption of the OEC and DD in the Search Engine, and (3) additional power to read from the SRAM and update the local-memory, where the impact of these steps on energy consumption is discussed earlier.

First, in our silicon photonics platform $E_{\text{dr}}>E_{\text{ht}}^{d,\max}$ , i.e., the driving power needed to re-modulate each of the bits of the data words in the TDM scheme is higher than to the heater power needed to store bits of *‘1’* in the WDM scheme. Indeed, a 1-tap driver in a 28 nm CMOS process, which we used in our previous work [13], has a power consumption per MRM of 6.075 mW. On the other hand, typical heaters have thermal tuning efficiency in the order of $20\;\;\frac{\text{mW}}{\text{FSR}}$ , which results in less than 0.5 mW to store *‘1’* in our microrings (FSR of 13.6 nm), when the channel spacing is set to 0.6 nm. This difference between driving power and static tuning is expected to be more significant for tuning using either MOSCAP [23] or non-volatile MRMs [40], as in these novel devices negligible power is required static tuning. Importantly, it is worth noting that this apparent disadvantage of the TDM O-TCAM scheme is linked to the specific circuit instantiation proposed in the previous sections of this paper. As we will explain with an example in Section 5.2, alternative TDM O-TCAM circuits can be proposed where the energy cost for driving the microrings to encode the data words can be amortized over multiple incoming search words, which should reduce the effective energy consumption of the driver per comparison significantly.

Second, for the TDM O-TCAM architecture, the powers of peripheral electronic circuits, i.e., TIA, comparator, and integrator, embedded in the OEC and DD devices are not shared between multiple bits as occurs for the WDM O-TCAM architecture. While the number of detected photons required to make an accurate decision will be similar for both WDM and TDM schemes, and consequently will not significantly change the required photon energy in each architecture, without dedicated adjustments of the electronic peripherals to the lower bandwidth requirements for the TDM architecture, where a Match/Mismatch only needs to be communicated at $\frac{R_{b}}{L}$ speed versus *R*_*b*_ for WDM, a power penalty for the TDM O-TCAM architecture arises. In addition to working with lower bandwidth electronics in the DD, a possible solution to share the power cost over multiple bits for the TDM O-TCAM circuit is using a PD that converts light to current only when the photon count has been above a predefined threshold during the duration of a word, which in our case differentiates between the Match and Mismatch states. As a result the peripheral electronic circuits can idle during the comparison process excluding minimal decision window after the end of the sequence, which expect to reduce their power consumption significantly. This PD specification can for instance be realized using similar functionality as what is currently obtained in single-photon avalanche diodes (SPAD) developed for quantum and biomedical applications [41, 42]. However, this device is not yet available in HPE’s fabrication platform. Therefore, we used our APD in the following energy consumption analysis, which will lead to a worst case estimate for the TDM O-TCAM architecture.

### 5.1 Energy estimation

**Figure 10:**
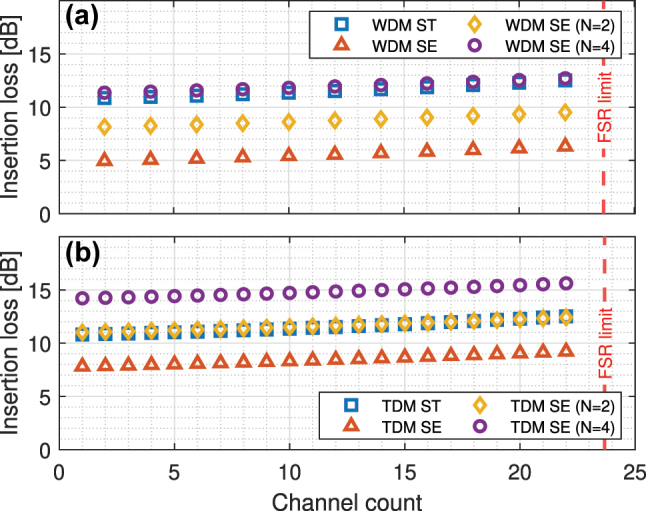
The end-to-end insertion loss of (a) a SiPh MRM-based transmitter, located in the WDM Search Transmitter (ST), and a SiPh MRM-based ML, located in the WDM Search Engine (SE) with single input, *N* = 2, or *N* = 4 as well as (b) a SiPh MRM-based transmitter, located in the TDM Search Transmitter, and a SiPh MRM-based cascaded ML, located in the TDM Search Engine with single input, *N* = 2, or *N* = 4.

The power consumption of the comb laser depends on the link insertion loss and the APD sensitivity. The SiPh components of the Search Transmitter and Search Engine are shown in Figure 3(b)–(c) for the WDM O-TCAM and in Figure 5(b)–(c) for the TDM O-TCAM. The insertion loss analysis is based on the design rules and performance of our exascale PDK. For readers convenience, key performance, which are used in the estimation of the energy consumption, of our PDK as well as supplemental decision-related devices are listed in Tables 2 and 3, respectively. The light of the comb source is coupled through a 2D grating coupler (GC), which interfaces with a 2000 μm medium-etch single mode (MESM) waveguide. The minimum loss and 1 dB bandwidth of the 2D GC is 2.6 dB (at 1310 nm) and 20 nm. Advanced design techniques, such as adjoint method-based shape optimization, are expected to improve the performance of 2D GC even further [43]. In the Search Transmitter and Search Engine, MRMs with FSR of 13.6 nm are cascaded along a deep-etch single mode (DESM) waveguide, where the channel spacing is 0.6 nm to ensure minimal crosstalk penalties in this very first performance analysis of O-TCAMs. In the future more detail analysis is required to study the impact of the channel crosstalk on the decision quality of the match signal, which can be estimated using similar methodology as in Ref. [44] or model-based simulation. We use PIN MRMs in our designs, where their through and drop ports losses are 0.1 and 3 dB, respectively. The pitch between the edge of DESM waveguide and its adjacent MRM, *p*_1_, is 15 μm, and the pitch between two adjacent MRMs, *p*_2_, is 225 μm. The losses of the MESM and DESM waveguides and medium-etch deep-etch (MEDE) taper are 1, 2 dB/cm, and 0.1 dB. The modulated light is transferred through a fiber array towards the Search Engine. Arrays of 1D GCs, which interface with a 50 μm MESM waveguide, and 2D GCs couple the light to and from the fiber array. The minimum loss and 1 dB bandwidth of 1D GC, where the light incidence angle is 0°, is 1.8 dB (at 1309 nm) and 21 nm. Better GC performance is achievable with larger incidence angle [45, 46]. In our analysis, the losses of the 1D and 2D GC connectors are 0.9 [46] and 1.8 dB, which we expect to be achievable in the near future. An optional *N*-way splitter, which is based on cascaded 1 × 2 multi-mode interferometer (MMI) that interfaces with MESM waveguide, is located at the front-end of the Search Engine to share the energy cost of the drivers and heaters of the Search Transmitter with multiple cascaded MRMs rows. The excess loss of our 1 × 2 MMI is 0.2 dB. The insertion loss performance of the Search Transmitter and Search Engine for the WDM and TDM schemes are summarized in Figure 10(a) and (b), respectively. The vertical red dashed line marks the FSR limit.

**Table 2:** Key performance of HPE exascale SiPh PDK.

Element	Parameter	Value
GC (1D, 0°)	Minimum loss^a^	1.8 dB
	Bandwidth (1 dB)	21 nm
	Connector loss	0.9 dB
GC (2D)	Minimum loss^b^	2.6 dB
	Bandwidth (1 dB)	20 nm
	Connector loss	1.8 dB
MRM (PIN)	FSR	13.6 nm
	Thru port loss	0.1 dB
	Drop port loss	3 dB
MMI (1 × 2)	Excess loss	0.2 dB
MESM waveguide	Loss	1 dB/cm
DESM waveguide	Loss	2 dB/cm
MEDE taper	Loss	0.1 dB
Comb laser	WPE	5 %
	Excess power	20 %
Driver	Power^c^	6.075 mW
Heater	Thermal tuning efficiency	20 mW/FSR
TIA	Energy efficiency	40 fJ/bit
APD	Sensitivity^d^	−22 dBm

^a^Minimum loss is at 1309 nm. ^b^Minimum loss is at 1310 nm. ^c^1-tap pre-emphasis in a 28 nm CMOS process. ^d^At TIA settings of 5 KΩ and 1 μA equivalent noise.

**Table 3:** Energy performance estimation of decision-related devices.

Element	WDM O-TCAM	TDM O-TCAM
Comparator	243 fJ/bit^a^	$\frac{243}{L}\;\;\text{fJ/bit}$
Integrator		90 fJ/bit^b^

For *R*_*b*_ = 10 Gbps. ^a^Strong-arm latch comparator in a 28 nm platform, where *V*_DD_ = 0.9 V and *C*_*P,Q*_, *C*_*X,Y*_ ≤ 200 fF. ^b^OpAmp with feedback capacitor is expected to consume 1 mA with 0.9 V.

**Figure 11:**
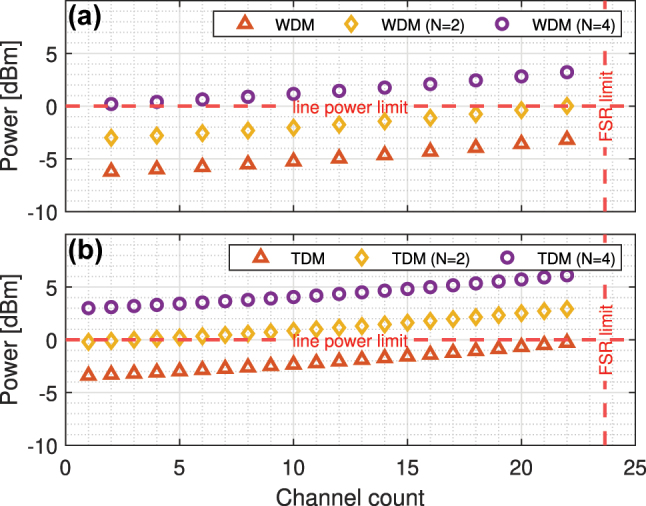
The required optical power of a comb line of (a) WDM Search Transmitter with single input, *N* = 2, or *N* = 4 as well as (b) TDM Search Transmitter with single input, *N* = 2, or *N* = 4.

In the determination of the required optical power of a single comb line, an APD from our exascale PDK was used followed by a TIA with gain of 5 KΩ and equivalent noise of 1 μA that translates to −22 dBm of required optical power at the APD input, which in SiPh Datacom/HPC interconnects corresponds to a BER of 10^−12^ [22]. The required optical power of a comb line, after considering the link insertion loss and OEC performance, for the WDM and TDM configurations, where the Search Engine has a set of one, two, or four *K* cascaded MRMs, are shown in Figure 11. The horizontal red dashed line at 0 dBm marks the power upper limit, which was selected to provide enough margin from the non-linear regime of the SiPh elements. The analysis shows that the splitter size is limited to 1 × 2, therefore at least $\frac{M}{2}\left(\frac{K}{2}\right)$ MRM-based transmitters are required to support *M* (*K*) entries of the Search Engine for the WDM (TDM) configuration. Moreover, the higher required power of a comb line in the case of TDM O-TCAM compared to WDM O-TCAM is a result of the difference in drop and through port losses in our microrings in HPE’s current PDK. However, this is a non-fundamental limitation of the TDM O-TCAM, as the difference between drop port versus through port insertion losses can be further minimized by reducing intrinsic waveguide losses or using different ring designs [47, 48]. A WPE of 5 % and excess power (EP) of 20 % was assumed in the obtaining of required electrical power of the comb laser [23]. Moreover, a 1-tap pre-emphasis 28 nm driver was considered, where its power consumption per MRM is 6.075 mW [13]. To align the microring resonance to its comb line, a post-fabrication thermal tuning of single channel spacing is required. In the energy analysis, we considered heaters thermal tuning efficiency of $20\;\;\frac{\text{mW}}{\text{FSR}}$ . Also, in the energy analysis of the WDM O-TCAM, an extra thermal tuning to statically position a microring at ‘1’ is used. The energy consumption of the TIA that goes after our APD is 40 fJ/bit [22, 23]. The DD circuity for both WDM and TDM configurations can be designed with low-power consumption. For example, strong-arm latch comparator is considered as energy-efficient design due to its zero static power consumption [25]. The power consumption of a strong-arm latch comparator is approximately $f_{\text{CK}}\left(2C_{\text{P,Q}}+C_{\text{X,Y}}\right)V_{\text{DD}}^{2}$ [25], where $f_{\text{CK}}=\frac{R_{b}}{2}$ is the Nyquist clock frequency, *V*_DD_ is the supply voltage, and *C*_*P,Q*_ and *C*_*X,Y*_ are the comparator capacitors. For 28 nm CMOS process, the supply voltage is 0.9 V and each of the capacitors of the strong-arm latch comparator is smaller than 200 fF. Therefore, the maximum energy consumption is 243 fJ/bit for the WDM O-TCAM architecture with decision speed of 10 Gbps. Because the decision speed in the TDM O-TCAM architecture is slower by a factor *L*, i.e., $\frac{R_{b}}{L}$ , the power consumption of the strong-arm comparator reduces to $\frac{243}{L}\;\;\text{fJ/bit}$ . Moreover, the integrating amplifier, required for the TDM O-TCAM architecture, can be realized by a low-power operational amplifier (OpAmp) with a feedback capacitor, which expected to consume 1 mA with 0.9 V. Alternatively, a capacitor-based integrator, similar to the design in Ref. [29], can be used, which is expected to improve substantially the power consumption compared to OpAmp integrator. However, once SPAD is available for CAM applications, the decision can be made at the end of the comparison sequence, and as a result the comparator can for instance be operated at idle mode the rest of the time to save power while the integrator is not required anymore. In our analysis, an OpAmp integrator is used to estimate the maximum energy of the decision circuitry.

**Figure 12:**
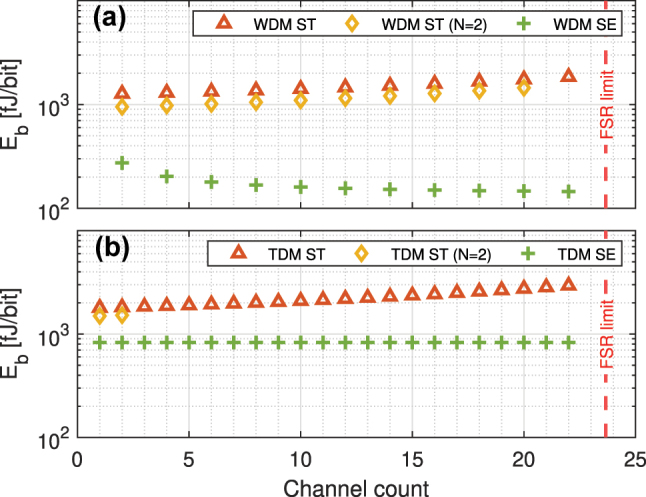
The energy consumption, excluding the SRAM and memory buffer for the TDM Search Engine, of the Search Transmitter (ST) and Search Engine (SE) of the (a) WDM O-TCAM as well as (b) TDM O-TCAM architectures. The channel count is equal to the number of cascaded microrings along a bus waveguide. In the case of WDM O-TCAM, each MRR stores one of the word bits, and in the case of the TDM O-TCAM, each MRM is being driven by one of the data words.

**Figure 13:**
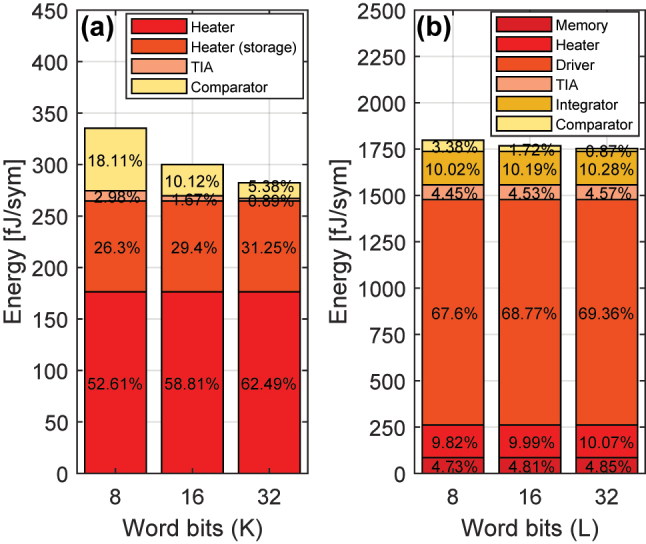
The contributions to energy efficiency from individual components of the Search Engine of the (a) WDM O-TCAM as well as (b) TDM O-TCAM architectures for various word bit count.

At a channel data rate of *R*_*b*_ = 10 Gbps, the energy consumption of the Search Transmitter and Search Engine variations are shown in Figure 12. In both architectures, the energy consumption of the Search Transmitter is significant due to the comb and drivers needed for the electro-optic conversion in current optical interconnect systems, which are tailored for E-CAMs. In the future, once all-optical interconnect systems, designed to be fully compatible with O-CAMs, are available the search signal can be extracted directly from the optical data stream, making the Search Transmitter redundant. The difference in the energy consumption of the Search Transmitter is a result of the different comb line power requirements between the two architectures, which is expected to be solved by engineering microrings with lower drop port losses. In the case of the WDM architecture, the energy cost of the Search Engine decreases as the length of the word increases, due to the reduction in contribution of the TIA and comparator to the total energy consumption, as predicted by the model in Eq. (6) and demonstrated in Figure 13(a) for *K* ∈ {8, 16, 32}. Significant reduction in this energy is expected in the near future, once the non-volatile microrings are mature to be included in our SiPh component library [23], thus making the thermal tuning redundant. Then the O-TCAM energy cost will be dependent only on the OEC and DD energy consumption divided by the channel count, and as a result our design will become competitive with low-energy E-TCAM designs [8, 49]. In the energy consumption of the TDM O-TCAM we assumed a word with 48 bits, as the length of an Ethernet MAC address. Therefore, *L* = 96 for the TCAM encoding scheme used in this work, which reduces the comparator energy consumption by the same factor, while the energy cost of the memory, driver, heater, TIA, and integrator is not affected by the word length as they operate at *R*_*b*_. This leads to Search Engine energy consumption of 1741.6 fJ/sym for ternary scheme (870.8 fJ/sym for binary scheme with *L* = 96). As predicted, the energy consumption of the TDM Search Engine is significantly higher than the WDM design, a result of the much higher driving power and microring drop loss as well as the mode of operation of the PD and DD. Moreover, as demonstrated in Figure 13(b) for a TDM O-TCAM architecture with *L* ∈ {8, 16, 32}, the driver substantially increases the total Search Engine energy consumption compared to the other engine devices. As discussed before, the energy cost of the proposed TCAM O-TCAM architecture can be reduce with design improvements in the microring, PD, and DD circuitry. However, the power arises from the multiple drivers, which are used to re-modulate each of the bits of the data words, cannot be mitigated in this specific instantiation of the TDM scheme and requires an alternative engine design, which will be presented in Section 5.2.

### 5.2 Alternative energy-focused TDM O-TCAM design

**Figure 14:**
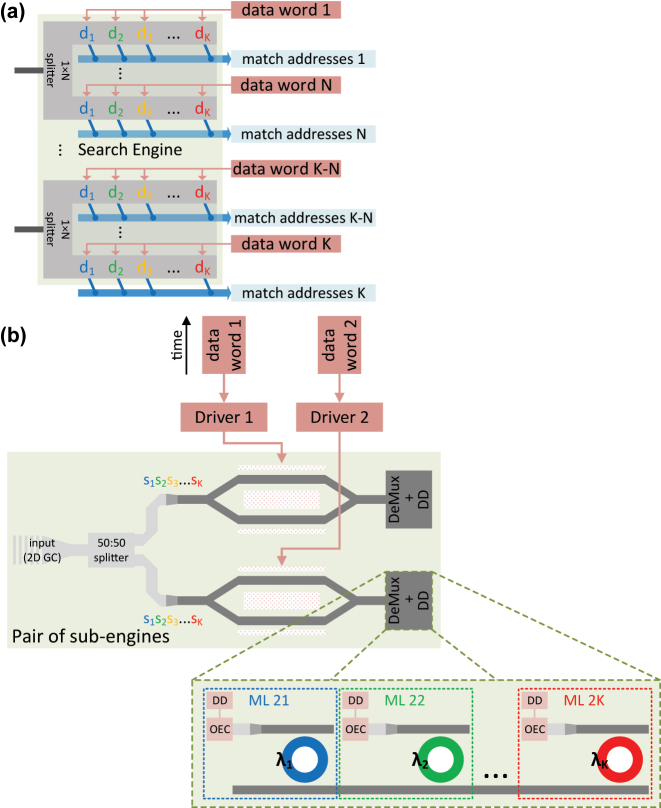
MZM-based TDM O-TCAM Search Engine architecture. (a) A block diagram of the alternative Search Engine architecture, where its core-operations of comparison and de-multiplexing are performed by the SiPh MZM and microring filters, respectively. (b) An example of pair of sub-engines, where the demultiplexer (DeMux) is based on add-drop microring filters. At the microring drop port the match signal is converted to electrical signal by the OEC and then the Match state is determined by the DD.

The proposed TDM O-TCAM architecture does not require serial-to-parallel word conversion, allows quick data word update, and has no FSR constraint on the word length, features in which the TDM scheme has an advantage over the WDM O-TCAM. However, the significant power consumed by the multiple drivers at TDM Search Engine limits its attractiveness compared to other CAMs. To address this energy burden, we propose here an alternative O-TCAM engine design that is inspired by the TDM schemes for AI applications in Refs. [44, 50]. Other photonics matrix-vector product architectures, e.g., free-space-based system [29], potentially offer better insertion loss scaling as a function of matrix size, but we focus here on pure integrated approach. A block diagram of the new Search Engine is shown in Figure 14(a). The search in this engine diagram is executed horizontally, i.e., wavelength domain, while in previous TDM scheme shown in Figure 5(a), the search is carried out vertically, i.e., spatial domain. Due to this modification, a row of cascaded MRMs can be replaced with a Mach-Zehnder modulator (MZM), which is driven by one data word, thus simplifying the electronic circuitry and considerably reducing the memory and driving energy cost. An example of sub-engine circuit is shown in Figure 14(b). After the *K* wavelength-encoded search words are multiplied by a data word through the re-modulation step, the results are demultiplexed and converted to electrical signal by cascaded microring filters followed by a PD. The matching state of each of the signals is determined by the DD. Therefore, for the alternative TDM O-TCAM design the energy consumption in Eq. (8) can be modified as:

(9) $$E_{\mathrm{SE}}^{\left(TDM\right)}=\frac{E_{\text{mr}}+E_{\text{dr}}^{\text{MZM}}}{K}+E_{\text{dd}}^{\left(TDM\right)},$$

where we assumed non-volatile MRRs for the demultiplexing. Similarly to the discussion in Section 5.1, SPADs can be used in this architecture for the OEC step, therefore, the match state can be determined at the end of the search sequence by a comparator. Idling the comparator for most of the match signal duration is expected to reduce the decision energy by a factor proportional to *L*, leading to a competitive energy consumption compared to the decision device in the WDM scheme. Furthermore, the power required to drive an MZM [51, 52] is higher than microring, however, the driving search energy in the new TDM architecture is reduced by a factor of *K* because one data word re-modulates *K* search words. Therefore, to fully leverage the energy saving potential of this TDM scheme, the number of words should be as high as possible, which can be achieved by using a wide-band comb laser with numerous lines [20], though, in this case the channel crosstalk and microring FSR constraints on the word count should be addressed. Cascading multiple stages of low-loss deinterleavers [53, 54] can alleviate the channel crosstalk. Moreover, by designing the comb line spacing such that the interference between the deinterleaved comb lines in different FSR sets is minimized, the effective transmission bandwidth can be increased significantly. As a result the total energy consumption of the new MZM-based TDM scheme is expected to be much lower than the non-volatile microring-based TDM architecture, but still higher than the WDM engine, unless additional methods can be found that reduce the driving power, $E_{\text{dd}}^{\left(TDM\right)}$ .

### 5.3 Energy performance comparison

To put our results in perspective, we compared the energy performance of our O-TCAM architectures with state-of-the-art E-TCAM and O-TCAM designs. Because competitive E-CAMs and O-CAMs report only on the engine performance, here, to compare apples to apples, we included only the engine-side. Moreover, it is worth to point out that the theoretic proposal of the WDM O-BCAM circuit proposed in Ref. [16] included an optimistic estimate of the minimal power consumption of such O-BCAMs to be 4.5 aJ/sym. based on the required *optical* power received at the PD at SNR = 1 for signal bandwidth of 50 GHz, which is linked to the laser power at the transmit-side (not yet including wall-plug efficiency of the laser, neither incorporates the effect of the link budget due to the losses in the passive devices between laser and PD). In contrast, for the comparison in this paper, we focus on the engine-side, i.e., the power related to the engine’s tuning of optical devices as well as required electronic circuitry, such as TIA and the comparator. Table 4 summarizes the comparison results between various TCAM engines. In the case of the WDM architecture, at a word length of 11 symbols, the energy consumption per ternary symbol is 290.4 fJ/sym, which is 2× the energy per channel, i.e., 145.2 fJ/channel at *K* = 22, because of the two-bit encoding scheme presented in Table 1. Note that when only O-BCAM functionality is required, following the proposed circuit in Ref. [16] where a single microring blocks light in one out of two incoming wavelength channels, the number of required microrings can be reduced by a factor 2, and the energy per symbol becomes 145.2 fJ/sym. When non-volatile microring are considered, the post-fabrication thermal tuning and thermal bit storing are redundant, which result in a power saving of 1.324 mW per heater, i.e., energy consumption of 25.8 fJ/sym (12.9 fJ/sym) for ternary (binary) symbol, an energy improvement by a factor of 11 compared to SiPh MRR-based WDM O-TCAM engine. In the case of the proposed MRM-based TDM O-TCAM engine, the energy consumption is 1741.6 fJ/symf for ternary symbols (see Section 5.1), six times higher compared to WDM O-TCAM engines. A significant reduction in the energy consumption of the TDM O-TCAM engine can be achieved when the SiPh MRMs are replaced with a SiPh MZM, as proposed in Section 5.2. Assuming our circuit would use the driver reported in Ref. [51], which has an energy consumption of 6.6 pJ/bit at 10 Gbps, then for a comb source with 135 lines [20] the energy consumption per word is 97.8 fJ/sym (48.9 fJ/sym) for ternary (binary) symbols. Moreover, if SPADs are being used for the OEC, then the TIA becomes redundant and the comparator can operate only at the end of the sequence and consequently its energy consumption reduces by at least a factor of *L*, the word length. At a word length of *L* = 48 (*L* = 96), the comparator energy consumption is 5.06 fJ/sym (2.53 fJ/sym) for ternary (binary) symbols. Therefore, the total energy consumption of this architecture is 16.8 times lower compared to MRM-based TDM O-TCAM engine.

**Table 4:** Energy performance comparison between various CAM engine technologies.

	E-TCAM			O-TCAM (O-BCAM)				
**Reference**	[49]	[11]	[7]	[55]	This work			
					At hand		In the near future	
					WDM	TDM	WDM	TDM
**Technology**	SRAM	Memristor	Memristor	InP SOA	SiPh MRR	SiPh MRM	MOSCAP MRR	SiPh MZM
**Method**	Experiment	Simulation	Experiment	Experiment	Simulation	Simulation	Simulation	Simulation
**Search speed**	400 MHz	1 GHz	100 MHz	10 Gbps	10 Gbps	10 Gbps	10 Gbps	10 Gbps
**Energy**	0.165 fJ/sym	0.17 fJ/sym	2.75 fJ/sym	300 pJ/sym (200 pJ/sym)	290.4 fJ/sym (145.2 fJ/sym)	1741.6 fJ/sym (870.8 fJ/sym)	25.8 fJ/sym (12.9 fJ/sym)	103.4 fJ/sym (51.7 fJ/sym)
**Included elements**	Driver, shifters, registers, etc.	Driver	Driver, TIA, ADC, comparator, etc.	SOA	Heater, TIA, comparator	Memory, driver, heater, TIA, comparator, integrator	TIA, comparator	Memory, driver, TIA, comparator
**Word length**	144 sym	36 sym	32 sym	4 sym	11 sym (22 sym)	48 sym (96 sym)	11 sym (22 sym)	48 sym (96 sym)
**Hamming distance**	No	No	Yes	No	Yes	Yes	Yes	Yes

## 6 Conclusions

In this work, we proposed two new SiPh microring-based O-TCAMs architectures—WDM O-TCAM and TDM O-TCAM. We demonstrated the operation of the proposed architectures through circuit simulation using our exascale silicon photonics PDK as well as lab experiments using a cascaded MRMs test structure. In both the simulation and experiment, we showed that in all cases the match signal follows the decision scheme of the proposed O-TCAM designs. Based on our exascale PDK design rules and performance, we analyzed the energy consumption of the proposed WDM O-TCAM and TDM O-TCAM. The analysis shows that the energy cost of the Search Engine of the WDM O-TCAM design reduces as the word length increases towards a minimum of 290.4 fJ/sym for ternary symbols. This energy consumption can be reduced by a factor of ∼10 when using either MOSCAP or non-volatile microrings [23, 40], which eliminate the power required for thermal tuning, to the energy consumption of the APD divided by the channel count. However, it should be taken into consideration that in this WDM scheme the word length is limited by the FSR. Moreover, the TDM O-TCAM, which in our initially microring-based proposed design has an energy consumption penalty of a factor of ∼6 compared to the MRR-based WDM O-TCAM scheme, due the additionally required drivers for the microrings at the search engine (i.e., receive) side. On the other hand, in the proposed design that is based on an MZM, this driver power can be amortized over multiple wavelength channels, reducing this penalty in a large channel count, e.g., an energy saving factor of 16 compared to MRM-based TDM O-TCAM for 135 channels, leading to 103.4 fJ/sym for ternary symbols. While E-TCAMs still have advantages in terms of energy consumption compared to O-TCAMs, our approach has the potential to lead to embedded O-TCAM circuits in the optical link and operate at signal data rate, therefore resolving current performance and latency bottlenecks. Furthermore, it is noteworthy to mention that the functionality of the proposed architectures goes beyond parallel content search, but has variations that are also capable of Hamming distance measurement, an important metric for similarity learning and hyperdimensional computing [7, 56].
